# *Alepidea amatymbica* Eckl. & Zeyh.: A Review of Its Traditional Uses, Phytochemistry, Pharmacology, and Toxicology

**DOI:** 10.1155/2014/284517

**Published:** 2014-08-17

**Authors:** O. A. Wintola, A. J. Afolayan

**Affiliations:** Medicinal Plant and Economic Development Research Centre, Department of Botany, University of Fort Hare, Alice 5700, South Africa

## Abstract

*Alepidea amatymbica* is an important medicinal plant in Southern Africa with a long history of traditional use for the management of conditions like colds, coughs, sore throat, influenza, asthma, and abdominal cramps. Despite the much acclaimed traditional uses of the plant, there is a dearth of scientific information on the review of this plant. Hence, this review is aimed at providing information on the botany, phytochemistry, pharmacology, and toxicology of *A. amatymbica*. This review uses all the synonyms of the plant obtained from the plant list. Google scholar, Science Direct, PubMed, and Scopus were made use of in addition to the University of Fort Hare's online databases. All the phytochemical studies on *Alepidea amatymbica* obtained from the literature reported the presence of kaurene-type diterpenoids and their derivatives. Pharmacological areas identified on *A. amatymbica* fresh and dried extract include antibacterial, antifungal, sedative, astringent, antimalarial, anti-inflammatory, antihelminthes, antihypertensive, anti-HIV, and diuretic activities. Literature search on *A. amatymbica* revealed the use of cell line, brine shrimps, and rats for the determination of the toxicity in the plant. Clinical trials and product development to fully exploit the medicinal value are also required to validate its folklore use in traditional medicine.

## 1. Introduction

The genus* Alepidea *is a member of the Apiaceae family, placed in the subfamily Saniculoideae, also known as the larger tinsel flower (Eng). Species from the genus have been historically important medicinal plants throughout Africa, occurring mainly in Southern Africa [1, 2].* Alepidea* is a genus of 28 species of herbaceous geophytes endemic to grassland areas of Eastern and Southern Africa northwards to Kenya and Ethiopia [1, 3, 4]. Seven out of the 28 species (*A. amatymbica* Eckl. & Zeyh.,* A. natalensis* Wood & Evans,* A. pilifera *Weimack,* A. longifolia* E. Mey. Ex Dümmer,* A. setifera *N.E.Br.,* A. comosa* Dümmer, and* A. macowanii* Dümmer) are known to be used commonly for medicinal purposes [5, 6]. Species of the genus* Alepidea *are most commonly found in the grasslands of the Eastern Cape Province, Kwazulu Natal, Mpumalanga, South Africa, and other Southern Africa countries like Lesotho, Swaziland, and Zimbabwe [7–9].

**Figure 1:**
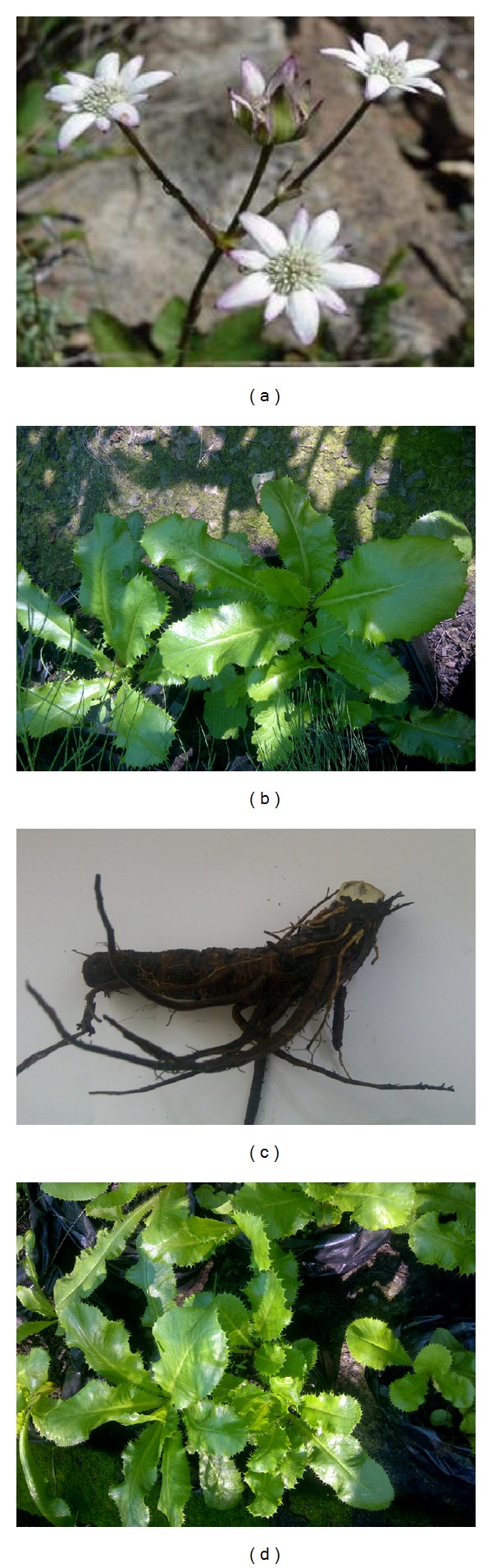
(a)* Alepidea amatymbica* Eckl. & Zeyh. in its natural habitat (source: http://www.Plantzafrica.com), (b) dried rhizome of* Alepidea amatymbica*, and ((c) and (d))* Alepidea amatymbica *growing in the nursery.

*Alepidea amatymbica* Eckl. & Zeyh. also known as kalmoes (Afr.); Iqwili (Xhosa); ikhathazo (Zulu) is an important source of traditional medicine [10, 11]. The plant generally grows on stream banks, drainage lines, and forest margins of Northern and Southern Drakensberg Mountains of the Eastern Cape Province, Lesotho, Kwazulu Natal, Swaziland, Mpumalanga, and Northern Province extending towards Zimbabwe [12]. The plant is critically endangered in Zimbabwe [13, 14], vulnerable in Lesotho [15], and listed as at lower risk or near threatened but locally extinct in certain heavily collected areas [14, 16].* A. amatymbica* is an herbaceous perennial plant with dark green leaves arising from a single or branched rhizome. It is a robust, erect plant, up to 2 m tall in grassland; the leaves form a loose rosette with the margins of the leaves prominently toothed, each tooth ending in a bristle. The flowering stalk hollow up to two meters in height, rising above the surrounding grasses, with numerous small flowers arranged in dense, rounded heads [8]. The inflorescence is widely branched, with a number of small, star-shaped white flowers about 250 mm in diameter (Figure 1).* A. amatymbica* has a number of synonyms, namely,* Alepidea amatymbica* var.* amatymbica*,* Alepidea amatymbica* var. cordata Eckl. & Zeyh.,* Alepidea aquatica *Kuntze, and* Eryngium amatymbica *(Eckl. & Zeyh.) Koso-Pol [17].

This review is aimed at gathering information on* Alepidea amatymbica* that covers its traditional use, phytochemistry, pharmacological activity, and toxicology and at highlighting the opportunities for greater development of the plant's medicinal properties at a local and international level.

## 2. Materials and Methods

During the review, all the synonyms of* Alepidea amatymbica* (*Alepidea amatymbica* var.* amatymbica*,* Alepidea amatymbica* var.* cordata* Eckl. & Zeyh.,* Alepidea aquatica *Kuntze, and* Eryngium amatymbica *(Eckl. & Zeyh.) Koso-Pol), derived from the plant list synonyms, http://www.theplantlist.org [17], were employed for the literature search. Key words such as “botany,” “pharmacological effects,” “toxicological effects,” and “traditional uses” of* Alepidea amatymbica* were used for the literature search. Searches were done on the following databases: Google Scholar (http://scholar.google.com), Science Direct (http://www.sciencedirect.com), PubMed (http://www.ncbi.nlm.nih.gov/pubmed), and Scopus (http://www.scopus.com).

## 3. Traditional Medicinal Uses

Various ranges of traditional uses of* Alepidea amatymbica* were reported in the literature, from simple uses for conditions such as malaria, diarrhea to cold, coughs, influenza, chest complaints, and wound to complex uses for the management of asthma and rheumatism. For asthma treatment, grounded samples of* Alepidea amatymbica* rhizome are combined with cannabis for the washing of the divining bones [18]. Our literature search identified citations for traditional use in six countries and these countries are located in Southern Africa (South Africa, Swaziland, Lesotho, and Zimbabwe) and East Africa (Kenya, Ethiopia). In traditional medicine,* Alepidea amatymbica* is used for the treatment of minor ailments (e.g., sore throat, cough, and influenza) and complications (inflammation, asthma, diarrhea, abdominal cramps, wound, and rheumatism). The ethnomedical uses of* Alepidea amatymbica* are listed in Table 1. In Zimbabwe,* A. amatymbica* is considered as one of the ten most recognised medicinal plants, locally used as a remedy for asthma, influenza, diarrhoea, and abdominal cramps and to treat colds, coughs rheumatism, and wounds [14, 19]. This species is highly regarded as a remedy for respiratory tract infections, asthma, sore throat, gastrointestinal complaints, fever, rheumatism, bleeding wounds, and headache and extracts are also reported to be active against HIV [2].

### 3.1. Posology (Dosage)

*A. amatymbica* is used for the treatment of colds and chest complaints Watt & Breyer-Brandwijk [10], as well as for asthma, influenza, diarrhoea and abdominal cramps, sore throat, and rheumatism [7, 12, 36]. For respiratory complaints (cough, cold, and influenza), the recommended adult dose is one tablespoonful of raw or cooked rhizome and root; for children one to two teaspoonfuls, according to age, is sufficient. Fresh or cooked rhizome and roots are chewed or sucked, and fresh rhizome is also applied externally as a styptic [7, 12]. The dry rhizome and roots are smoked or powdered and taken as a snuff by unspecified diviners and healers in South Africa to assist in divination and communication with the ancestors [20]. Smoke from burning of the dry plant material is inhaled and a root infusion taken orally or administered per rectum as an enema [25]. Smoking the roots results in mild sedation and vivid dreams, and a decoction made of the dried product is taken or fresh rhizomes are chewed. It is also burned and inhaled or administered as snuff [7, 12]. Zulu herbalists (Izinyanga) prescribe the plant to help prevent nervousness in South Africa [25].

### 3.2. Contradictions

*Alepidea amatymbica* rhizome is believed to be used as a diuretic by Somova et al. [24], but the plant was reported by Wright et al. [37], to have no diuretic effect in an overview of plants with putative diuretic effects.

## 4. Chemical Constituents

**Figure 2:**
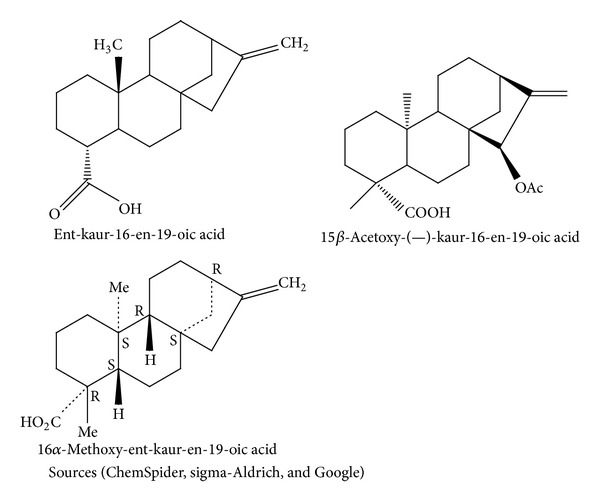
Chemical structures of kaurene-type diterpenoids in* Alepidea amatymbica.*

Some of the phytochemicals isolated and characterized to date from* Alepidea amatymbica* are showed in Figure 2. These include kaurene-type diterpenoids and their derivatives like ent-9, (11)-dehydro-16-kauren-19-oic acid, ent-16-kauren-19-oic acid, wedelia seco-kaurenolide, and 313-acetoxy which is believed to constitute up to 11.8% of rhizome and root dry mass [20]. The activity of the medicine can most likely be attributed to the diterpenoids it contains, although they have not been tested individually [20]. Several diterpenoid kaurene derivatives have been isolated from the rhizomes and roots of* A. amatymbica* [30, 31, 38]. Lipophilic extracts of the powdered dried rhizomes of* A. amatymbica* collected in different localities also confirmed the presence of ent-9, (11)-dehydro-16-kauren-19-oic acid (la), ent-16-kauren-19-oic acid (2a), wedelia seco-kaurenolide (3) and (4), and the 313-acetoxy derivative of 3, previously reported as constituents of the roots and aerial parts of* A. amatymbica* [30]. The extracts contained additional kaurene derivatives not previously reported. A comparative study of dichloromethane extracts of the roots of several* Alepidea *species showed the presence of kaurene derivatives in every case. The distribution of all the major compounds found in* Alepidea amatymbica* is summarized in Table 2. The highly resinous rhizomes contain kaurene-type diterpenoids [30, 31].

## 5. Pharmacological Reports

Different pharmacological areas were revealed in the literature search on* A. amatymbica* investigation. These include anti-inflammatory, antibacterial, antifungal, antihelmintic, antimalarial, antihypertensive, and diuretic activities. The summarized detail of the pharmacological studies on* A. amatymbica* was showed in Table 3. However, there is dearth of information in literature on the pharmacological effects of the active principles of* A. amatymbica*. Hutchings [27] reported the performance of screening tests on the basis of personal communication with a pharmaceutical company indicating the antimicrobial, antihypertensive, and diuretic activity [24].

### 5.1. Antimicrobial Activity

Crude dichloromethane, petroleum ether, ethanol, and aqueous rhizome extracts demonstrate strong antibacterial activity against both gram-negative and gram-positive bacteria used. However, the crude PE and DCM rhizome extracts exhibited the best MIC (0.39 mg/mL) against* B. subtilis*, and the rest of the extracts were shown to have low activity (MIC value > 1 mg/mL) [6]. The dosage and MIC of the plant extract used in this study showed activity against the bacterial tested when compared to the control. This suggests that the result favors its usage in traditional medicine for the treatment of bacterial infections.

In a related work, the crude extracts of the leaf, stem, rhizome, and root of* A. amatymbica* exhibit a range of antimicrobial properties against the following bacteria:* Bacillus cereus, Staphylococcus epidermidis, Staphylococcus aureus, Micrococcus kristinae, Streptococcus pyogenes, Escherichia coli, Salmonella poona, Serratia marcescens, Pseudomonas aeruginosa, and Klebsiella pneumoniae.* The acetone rhizome extract showed better activity than others especially on* S. aureus and B. cereus* and moderate activity was recorded against the gram-positive bacteria tested with the exception of* Micrococcus kristinae *[8]. Although the inhibition was high in all the parts of the plant tested, the acetone and methanol stem extract showed a weak activity against* Streptococcus pyogenes*. This result supports the use of* A. amatymbica* in treating bacterial diseases associated with respiratory tract, urinary tract, and gastrointestinal tract infections. Due to lack of positive control in this work, it is difficult to draw conclusions on the study. Thus further investigation is required to justify its traditional usage as antibacterial.

The fresh and 90-day dried crude extract of the leaf and rhizome extract of* A. amatymbica* prepared in water, ethanol, and hexane were used in this study [9]. The extracts demonstrated anti-*H. pylori* activity with zone of inhibition range of 0–38 mm and MIC_50_ range of 0.06–5.0 mg/mL, respectively. The antimicrobial activity was comparable with the control antibiotics. However,* A. amatymbica* extracts gave a susceptibility of less than 50%.

The antibacterial activity also increased with storage or ageing of plant material [33]. Antibacterial activity is stable in dry specimens and, as such, may account for the fact that activity is unaffected by storage in certain instances [39]. Therefore, the result favors the use of the plant as an antibacterial considering the activity of the extract and the control even though the dosage was not stated. Hence more work is needed in the validation of the plant extract for the treatment of bacterial infections.

### 5.2. Antifungal Activity

Antifungal activities of* A. amatymbica* have been reported [6, 8]. The crude extracts of the leaf, stem, rhizome, and root exhibited activity against* Candida albicans* extracts and more than 50% mycotic inhibition against fungal cultures [6]. This study does not provide a dose dependent evidence of the extract and negative control was not used in validating the traditional use of the plant as antifungal. Also, all the extracts except acetone extract showed mycotic inhibition activity against* Aspergillus flavus* and* Aspergillus niger* [8]. These results validate the traditional uses of the plant as an antifungal but no dose dependent evidence was given in this study and positive control drug was not used in comparison with the extract used. Therefore, the antifungal activity of the extract cannot be compared with an antifungal drug.

### 5.3. Antiviral

Louvel et al. [2], investigated the screening activity of the aerial part and root aqueous extract of* A. amatymbica* against a cell-based infection assay targeting the replication of prototypic CXCR4-tropic (NL4-3) or CCR5-tropic (NL-AD87) HIV-1 strains designed to detect inhibitors blocking any step of the viral life cycle. The active ingredient identified in the extract does not support a direct application of this plant extract for treating HIV infection. The anti-HIV activity of the pure compound was found to be quite moderate. Lack of negative control and a dose dependent activity of the extract make the antiviral activity of the extract inconclusive.

### 5.4. Antihelminthes

Research was undertaken using nematode growth agar with* Caenorhabditis elegans *var. Bristol (N2)nematodes on the ethanol extract of the fresh and stored leaves and root of* A. amatymbica.* Only fresh and stored water extracts showed a significant Anthelminthic [33]. The result of the study did not evaluate a dose dependent inhibition of the extract against the worms, even though the controls were compared with the extract. Further investigation is thus required to validate the use of the plant in traditional medicine as antihelmintic.

### 5.5. Anti-Inflammatory

The DCM, PE, water, and EtOH rhizome extracts were evaluated using the enzyme based cyclooxygenase assays COX-1 and COX-2 [6]. The PE and DCM extracts had high COX-1 and COX-2 activities with percentage inhibitions above 70%. Ethanol extracts showed higher inhibition (<40%) in COX-1 than COX-2. The water extract on the other hand has a moderate inhibition activity (40–60%) in COX-1 and lowered in COX-2 (<20%). Despite reports on the undesirable effects of higher COX-1 inhibition as a result of its damage to the gastrointestinal tract [40], report of Mulaudzi et al. [6] recorded a higher COX-1 in both DCM and PE and a lower COX-1 inhibition in ethanol and water. The result favors the use of water and ethanol as a solvent of extraction for this plant when using it against inflammation. However, conclusion cannot be totally drawn on this report due to lack of negative control and dosage. Hence further investigation is required to provide an evidence for its traditional use against inflammation.

### 5.6. Antihypertensive

Hexane extractive (AA/1), dichloromethane extractive, and methanol extractive of the fresh rhizome were investigated using purified compounds on blood pressure and heart rate of anesthetized Wistar rats. Moderate, but significant, decrease in systolic blood pressure (SBP) and heart rate (HT) effects after intraperitoneal application on conscious rats was reported [24].

### 5.7. Antiplasmodium

Dichloromethane (DCM), DCM/methanol (MeOH) (1 : 1), MeOH, and purified water extract from the whole plant were investigated on plasmodial activity and were found to contain plant based antimalarial agents showing promising antiplasmodial activity with IC_50_ values of ≤10 g/mL [34]. This is the only study showing the dose dependent antiplasmodic activity of the plant extract in relation to the positive control. Validation of the use of the plant in vivo is necessary to conclude the antiplasmodial activity of* A. amatymbica.*

### 5.8. Diuretic

The purified compounds of the fresh rhizome extracted in hexane/ethyl acetate of* A. amatymbica *were investigated using anesthetized Wistar rats. The diuretic and natriuretic effects of the extractives were found to be similar to the effects of chlorothiazide drug [24]. The result suggests the inhibitory effect of the extractive of* A. amatymbica* in reabsorption of K^+^ and Na^+^ ions suggesting the diuretic and natriuretic effects of the extractives. Lack of tested extractive in a dose dependent manner however negates the validation of the plants and the extractives for use as diuretic traditionally. Further investigation is required to provide evidence for its use as a diuretic.

## 6. Toxicological Reports

Four toxicity screens were identified in the literature. Each of the four screens tested uses crude aqueous and organic solvents, hexane extracts, and extractives from the crude hexane extract and none of the reports recorded toxicity at all doses tested. The reported cytotoxicity of the fresh aqueous rhizome was investigated using HeLa, Vero, and Jurkat E6.1, AA-2, or CEM-SS cells. The extract was shown not to be toxic at all concentrations used in the test [35], since the dosage and controls were shown in this study in comparison with the control. The second screens use bacterial cultures (100 *μ*L) in an Amen test assay which were added to 100 *μ*L of DCM, PE, water, and EtOH rhizome plant extracts in 500 *μ*L phosphate buffer and 2 mL of agar containing biotin-histidine (0.5 mM). The Ames test revealed that none of the plant extracts significantly increased the number of His+ revertants with respect to the negative control [6]. The third test screen utilised the Brine Shrimp Lethal Assay (BSLA). Acute toxicity of the isolates from* Alepidea amatymbica* was screen against brine shrimp* Artemia salina* intersection bioassay. The brine shrimp test showed that the crude hexane extracts have low toxicity with LC_50_ 0.2 ng/mL, while the AA6 (wedelia seco-kaurenolide) a derivative of* A. amatymbica* has a low toxicity of 0.5 ng/mL [24]. The fourth screen made use of Hippocratic test on rats with the use of hexane (AA/1) extract and it's extractives in dichloromethane and methanol (AA/1) after subjection to repeated flash chromatography with gradient elution (100–70% hexane/EtOAc) to give AA/3, a crystalline mixture of ent-kaur-en-19-oic acid, ent-kaura-9 (11), 16-dien-19-oic acid, and trachyloban-19-oic acid, AA/4, 16-methoxy-ent-kaur-11-en-19-oic acid, AA/5, 11-acetoxy-ent-kaur-16-en-19-oic acid, and AA/6, wedelia seco-kaurenolide. All extracts, crude and purified, showed low toxicity ranging from 0.5 to 5 ng/mL (LC_50_) apart from AA/4 and AA/5 that produced slight diarrhea on days 3, 4, and 5 and other extractives showed no toxicity at a dose of 20 [24]. Apart from the studies of Treurnicht [35] and Mulaudzi et al. [6] mentioned above, the other studies do not provide sufficient evidence with respect to the safety of* A. amatymbica* considering the absence of dosage used and control in their assays.

## 7. Clinical Trials

To date there appears to be no published research indicating that extracts of* Alepidea amatymbica* have undergone human clinical trials.

## 8. Conclusion

The review showed that* A. amatymbica* has a widespread use in South Africa and other SADEC countries. The recognition of its ethnomedicinal usage for conditions of inflammation like rheumatism and sore throat to usage for wounds, cough, asthma, influenza, diarrhea, stomach cramps, abdominal disorders, malaria, and diuretic is worth mentioning. The ability of the extract to also restrain the growth of bacteria and fungi indicated its broad spectrum antimicrobial prospective. The pharmacological reports on* A. amatymbica* revealed therapeutic potential in the treatment of inflammation, malaria, and infectious diseases like influenza, cough, and diarrhea. The frequently occurring chemical constituents of* Alepidea amatymbica* belong to the kaurene-type diterpenoids and their derivatives. Literature search on* A. amatymbica* revealed the use of cell line, brine shrimps, and rats for the determination of the toxicity in the plant. More investigations are necessary to explore the medicinal potential of the plant in the management of rheumatism, asthma, hypertension, diuretic, and inflammation. Clinical trials and product development to fully exploit the medicinal value are also required to validate its folklore use in traditional medicine. There is therefore the need to develop the existing traditional use of this plant to amplify nutraceuticals product and commence clinical research to exploit the plant's novel phytochemicals.

## Figures and Tables

**Table 1:** Traditional uses of *Alepidea amatymbica* Eckl. & Zeyh.

Category of use	Description of traditional dosage	References
Cultural and dietary	The dry rhizome and roots are smoked or powdered and taken as a snuff by diviners and healers to assist in divination and communication with ancestors	[20–22]
	Smoking the roots reportedly results in mild sedation and vivid dreams	[22]
	The dry rhizome and roots are used as a lotion to wash the divining bones	[23]

Antihypertensive	Fresh rhizomes	[22, 24]

Nervousness	Dry rhizome and roots	[25]

Antimicrobial	Leaf, stem, rhizome, and root	[8, 22]

Diuretic effects	Fresh rhizome	[22, 24]

Respiratory	Rhizomes and roots are used for colds, coughs, and influenza and respiratory ailments	[7, 10, 21, 22, 25, 26]

Inflammatory conditions	Rhizomes and roots are used for rheumatism and wounds	[10, 21, 22]

Gastrointestinal	Rhizomes and roots are used for stomach	[10, 21, 22]

Purgative	Rhizomes for the treatment of abdominal disorders	[7, 27]

Mild sedation and vivid dreams	Smoking the roots	[20, 28]

Antimalaria	Rhizome	[29]

Astringent	Fresh rhizome is applied externally	[7, 12]

**Table 2:** Chemical groups, part of the plant studied, and isolated compounds isolated from *Alepidea amatymbica* Eckl. & Zeyh.

Phytochemicals	Compound	Plant part	Reference
Terpenes (kaurene-type diterpenoids)	ent-9, (11)-dehydro-16-kauren-19-oic acid	Rhizomes and roots	[30, 31]
	ent-16-kauren-19-oic acid	Dried rhizomes	[30]
	wedelia seco-kaurenolide	Dried rhizomes	[12, 30]
	313-acetoxy	Dried rhizomes	[30]

Phenolic acid	Phenolic acid	Rhizome	[32]

Rosmarinic acid	3′-O-*β*-d-Glucopyranosyl rosmarinic acid	Rhizome	[2]

**Table 3:** Pharmacological investigation of *Alepidea amatymbica* Eckl. & Zeyh.

Activity tested	Model used	Plant part used/tested material	Extract type	Dosage	Control	Results	Reference
Antibacterial	Nutrient agar medium was mixed with extract and bacteria suspension. Species strains: *Bacillus subtilis* ATCC 6051 and *Staphylococcus aureus* ATCC 12600 and *Escherichia coli* ATCC 11775 and *Klebsiella pneumoniae* ATCC 13883	Rhizome	DCM, PE, water, and EtOH rhizome extracts	1 mg mL	Water and bacteria free broth (−ve), 0.1 mg/mL neomycin (+ve)	The PE and DCM extracts of the rhizomes exhibited the best activity (MIC values of 0.39 mg/mL) against *B. subtilis*. The rest of the extracts showed low activity (MIC values >1 mg/mL)	[6]

Antibacterial	Agar dilution methods with the following organisms: *Bacillus cereus, Staphylococcus epidermidis, Staphylococcus aureus, Micrococcus kristinae, Streptococcus pyogenes, Escherichia coli, Salmonella poona, Serratia marcescens, Pseudomonas aeruginosa, and Klebsiella pneumoniae *	Crude extracts of the leaf, stem, rhizome, and root	Acetone and methanol	1–10 mg/mL	Plates containing 1% acetone and methanol in agar	The acetone rhizome extract showed better activity than others especially on *S. aureus *and* B. cereus* moderate activity that was recorded against the gram-positive bacteria tested with the exception of *Micrococcus kristinae *	[8]

Antimicrobial	Microdilution method on Mueller-Hinton broth. Species *Bacillus subtilis, Staphylococcus aureus, Escherichia coli,* and *Klebsiella pneumoniae *	Rhizome and leaf (fresh, 90 days old, and one-year- old material) were assayed	Water, ethanol, and hexane extracts	12.5, 6.25, 3.13, 1.56, 0.78, 0.39, 0.20, and 0.1 mg/mL	Extract-free solution and ethanol were used as a blank control and neomycin (+ve)	The water extracts of plants tested for antibacterial activity showed no activity, whereas the ethanol extracts generally showed an increase in activity. The antibacterial activity increase with storage or ageing of plant material	[33]

Antimicrobial	Agar well diffusion method using *H. pylori* inocula prepared at McFarland's turbidity standard 2 was plated onto BHI agar supplemented with 5% horse blood and Skirrow's supplement	Roots/rhizomes	Ethyl acetate, acetone, ethanol, methanol, and water	100 mg/mL	Clarithromycin and 10% DMSO were used as positive and negative controls, respectively	The plants demonstrated anti-*H. pylori* activity with zone diameters of inhibition between 0 and 38 mm and 50% minimum inhibitory concentration (MIC50) values ranging from 0.06 to 5.0 mg/mL	[9]

Antimicrobial	Agar well diffusion method. *Alepidea amatymbica* were investigated against 30 clinical strains of *H. pylori *	Roots/rhizomes	Ethyl acetate, acetone, ethanol, methanol, and water	0.002–5.0 mg/mL	Reference control strain (NCTC 11638). Metronidazole and amoxicillin were included as positive control antibiotics	Methanol was quantitatively the best solvent for all the plants while ethyl acetate had the lowest yields. *A. amatymbica* gave percentage susceptibilities of less than 50%	[9]

Antifungal	Agar dilution methods with the following organisms: *Aspergillus flavus, Aspergillus niger*, and *Penicillium notatum* cultures were maintained on Potato Dextrose agar	Crude extracts of the leaf, stem, rhizome, and root	Acetone and methanol	Radial pattern of streaking of organisms was used	Plates containing only PDA or PDA with the respective solvent	Diameter of the fungal growth was measured and expressed as percentage growth inhibition. All the extracts showed more than 50% mycotic inhibition with activity ranging from 51.39% to 81.11% at 5 mg/mL with the rhizome	[8]

Antifungal	The antifungal activity of the extracts was evaluated against *Candida albicans* (ATCC 10231) and fungal culture was prepared in Yeast Malt (YM)	Rhizome	DCM, PE, water, and EtOH rhizome extracts	0.1–5.0 mg/mL	Water and bacteria free broth (−ve), 0.1 mg/mL neomycin (+ve)	All the extracts showed activity against *Candida albicans *	[6]

Anti-inflammatory	Using the enzyme based cyclooxygenase assays COX-1 and COX-2	Rhizome	DCM, PE, water, and EtOH rhizome extracts	1 mg mL	Water and bacteria free broth (−ve), 0.1 mg/mL neomycin (+ve)	The PE and DCM extracts had high COX-1 activity with percentage inhibitions above 70%. Ethanol extracts had inhibition less than 40%	[6]

Antihypertensive	Purified compounds on blood pressure and heart rate of anesthetized Wistar rats.	Fresh rhizomes	Hexane extractive (AA/1), dichloromethane extractive, and methanol extractive	i.p. injection of sodium thiopentone (40 mg/kg body weight)	Chlorothiazide	In addition to the cardiovascular effects, distinct diuretic and natriuretic effects were found	[24]

Antiplasmodium	*Plasmodium falciparum* strain D10 using the parasite lactate dehydrogenase (pLDH) assay	Whole plant	Dichloromethane (DCM), DCM/methanol (MeOH) (1 : 1), MeOH, and purified water	100–0.2 *μ*g/mL)	Chloroquine diphosphate served as the positive control	Detect plant-based antimalarial agents showing promising antiplasmodial activity with IC50 values of ≤10 g/mL	[34]

Diuretic	The Lipschitz test was used to determine the Diuretic and saluretic activity in rats	Fresh rhizome	Hexane/ethyl acetate extract	The test compound was applied orally at a dose of 50 mg/kg	Urea (1 g/kg b.w.) Hydrochlorothiazide (25 mg/kg b.w.)	The diuretic and natriuretic effects of the extractives were found to be similar to the effects of chlorothiazide	[24]

Cardiovascular	Purified compounds on blood pressure and heart rate of anesthetized Wistar rats	Purified compound from fresh rhizome	Hexane extract (AA/1), dichloromethane, and methanol portion of AA/1 were subjected to repeated flash chromatography with gradient elution (100–70% hexane/EtOAc) to give AA/3, a crystalline mixture of ent-kaur-en-19-oic acid, ent-kaura-9 (11), 16-dien-19-oic acid, and trachyloban-19-oic acid, AA/4, 16-methoxy-ent-kaur-11-en-19-oic acid, AA/5, 11-acetoxy-ent-kaur-16-en-19-oic acid, and AA/6, wedelia seco-kaurenolide	20 mg/kg b.w. intraperitoneally	Ethylene glycol	Moderate, but significant, decreasing systolic blood pressure (SBP) and heart rate (HT) effects after intraperitoneal application on conscious rats	[24]

Anti-HIV	Extracts and therein subfractions of *A. amatymbica* were assessed in a cell based assay targeting the replication of prototypic CXCR4-tropic (NL4-3) or CCR5-tropic (NL-AD87) HIV-1 strains	Aerial parts and roots	Aqueous	500 *μ*L of sample at a concentration of 25 mg/mL	Standard retroviral inhibitor	The active ingredient identified in the aqueous extract does not support a direct application of this plant extract for treating HIV infection. The anti-HIV activity of the pure compound was found to be quite moderate	[2, 12]

Antihelminthes	Nematode growth agar with *Caenorhabditis elegans* var. Bristol (N2) nematodes	Fresh and stored leaves and root	Ethanol	1 mg/mL	Levamisole (+Ve) and nematode incubated with water (−Ve)	Only fresh and stored water extracts showed a significant antihelminthes	[33]

Toxicity	The bacterial cultures (100 *μ*L) were added to 100 *μ*L of plant extract in 500 *μ*L phosphate buffer and 2 mL of agar containing biotin-histidine (0.5 mM)	Rhizome	DCM, PE, water, and EtOH rhizome extracts	50, 500, and 5000 *μ*g/mL	4-Nitroquinoline-N-oxide (4NQO) was used as a positive control and water (−ve control)	The Ames test revealed that none of the plant extracts significantly increased the number of His+ revertants with respect to the negative control	[6]

Cytoxicity	HeLa, Vero, Jurkat E6.1, AA-2, or CEM-SS cells	Fresh rhizomes	Aqueous	1 mg/mL	Not stated	The extract was not toxic at any concentration used in the test	[35]

Acute toxicity	Evaluation using brine shrimp *Artemia salina* intersection bioassay	Fresh rhizomes	Hexane	Not stated	Not stated	The brine shrimp test showed that the crude hexane extracts have low toxicity with LC_50_ 0.2	[24]

Toxicity	The Hippocratic test on rats was used	Fresh rhizomes	Isolates from *Alepidea amatymbica* Hexane extractive	0.1 mg/mL	Verapamil (10 mg/kg)	All extracts, crude and purified, showed low toxicity ranging from LC_50_ 0.5 to 5 ng/mL apart from AA/4 and AA/5 that produced slight diarrhea on days 3, 4, and 5 and other extractives showed no toxicity at a dose of 20	[24]
